# Sporogony and sporozoite rates of avian malaria parasites in wild *Culex pipiens pallens* and *C. inatomii* in Japan

**DOI:** 10.1186/s13071-015-1251-1

**Published:** 2015-12-15

**Authors:** Kyeongsoon Kim, Yoshio Tsuda

**Affiliations:** Joint Department of Veterinary Medicine, Faculty of Agriculture, Tottori University, 4-101 Koyama-cho Minami, Tottori, 680-8553 Japan; Department of Medical Entomology, National Institute of Infectious Diseases, Toyama 1-23-1, Shinjuku-ku, Tokyo, 162-8640 Japan

**Keywords:** Avian malaria, *Culex inatomii*, *Culex pipiens pallens*, Natural vector, *Plasmodium*, Sporozoite rate, Vector competence

## Abstract

**Background:**

Malaria infection in mosquitoes is traditionally detected by microscopic examination for *Plasmodium* oocysts and sporozoites. Although PCR is now widely used, the presence of parasite DNA in a mosquito does not prove that sporogony is achieved. Thus, detection of sporozoites by microscopy is still required to definitively identify vector mosquitoes. The aim of this study was to confirm sporogony of avian *Plasmodium* spp. in *Culex pipiens pallens* and *C. inatomii* caught from the wild.

**Findings:**

Mosquitoes collected at two sites in Japan were dissected and examined by microscopy for *Plasmodium* oocysts and sporozoites. DNA was extracted from the midgut and salivary gland of infected mosquitoes, and the infecting *Plasmodium* species was identified by sequencing 478 bp of cytochrome *b*. Oocysts, or both oocysts and sporozoites, were found in 3.94 and 0.46 % of *C. p. pallens* and *C. inatomii*, respectively. Four (CXPIP09, GRW4, GRW11 and SGS1) and three cytochrome *b* lineages (CXINA01, CXINA02 and CXQUI01) were confirmed to achieve sporogony in *C. p. pallens* and *C. inatomii*, respectively. One mosquito each of *C. p. pallens* and *C. inatomii* was co-infected with two different *Plasmodium* lineages.

**Conclusions:**

These findings demonstrate that *C. p. pallens* and *C. inatomii* are natural vectors of four and three lineages of avian *Plasmodium* spp., respectively. The data indicate that a systematic procedure combining microscopy and PCR is a feasible and reliable approach to identify natural vectors of wildlife malaria.

## Findings

### Background

Malaria parasitization in mosquitoes is traditionally detected by dissection and microscopic examination for oocysts in the midgut and sporozoites in the salivary gland [1–3]. However, many recent field studies have relied on PCR instead [4–6]. Nevertheless, detection of *Plasmodium* DNA in a blood-sucking insect does not prove that the insect acts as vector [7, 8], as parasites are eliminated in a refractory insect. Thus, while PCR is sensitive enough to detect DNA from degraded parasites, microscopic detection of sporozoites remains necessary to verify sporogony and to identify competent vectors. On the other hand, oocysts and sporozoites vary little in morphology across *Plasmodium* species, and are impossible to identify to species or lineage by microscopy [9, 10]. Thus, a combination of dissection and PCR is required [5, 10, 11]. Unfortunately, this combined approach has not been adopted, except in studies of human malaria parasites.

The aim of this study was to use this combined approach to definitively establish whether *Culex pipiens pallens* and *C. inatomii* are competent vectors for avian malaria. Although these mosquitoes have been suggested in PCR-based studies to be primary natural vectors of avian malaria in Japan [11–13], sporogony has not been confirmed. Our results suggest that a systematic procedure combining dissection and PCR is a reliable approach to identify natural vectors of wildlife malaria.

### Methods

Mosquitoes were collected in Rinshi-no-mori park (35°37′ N, 139°42′ E) in Tokyo and Sakata wetland (37° 49′ N, 138° 53′ E) in Niigata, Japan, where transmission of multiple avian malaria parasites has been detected by PCR [11, 13]. The study sites and the ecological differences between *C. p. pallens* and *C. inatomii* are described in greater detail in our previous publications [11, 13]. In Rinshi-no-mori park, mosquitoes were collected once or twice a week from May to September in 2012 and from May to June in 2013, using a sweep net 36 cm in diameter as previously described [13]. In Sakata wetland, mosquitoes were collected on 2–3 July 2013 and on 30 June and 1 July 2014 using ten battery-operated suction traps (Inokuchi-Tekko, Nagasaki, Japan) baited with dry ice. The traps are similar to devices designed by the Centers for Disease Control and Prevention. Mosquitoes collected from the field were kept alive until dissection at National Institute of Infectious Diseases in Tokyo and Tottori University in Tottori.

Mosquitoes were immobilized by chilling or by chloroform, dissected according to WHO protocols [1], and examined under a microscope. The midgut was first examined for oocysts, and, when oocysts were present, the midgut and a part of the salivary gland were transferred to a 1.5 ml tube for DNA extraction. In addition, a smear of the salivary gland was stained by Giemsa and carefully examined for sporozoites.

DNA was extracted using REDExtract-N-Amp PCR Reaction Kit (Sigma Chemical Co., St. Louis, MO). A 478 bp fragment of *Plasmodium* cytochrome *b* was amplified by nested PCR according to Waldenström et al. [14], with slight modification [7]. Amplification products were purified with QIAEX II-Gel Extraction Kit (QIAGEN), and sequenced in both directions on an ABI PRISM 3130 Genetic Analyzer (Applied Biosystems), using ABI PRISM BigDye Terminator Cycle Sequencing Kit version 1.1 (Applied Biosystems, Foster City, CA, USA). Sequences were analyzed in GENETYX-WIN ver. 11, and compared to published sequences by a BLASTn search against the NCBI GenBank database and MalAvi database [15]. Sequences from one specimen each of *C. p. pallens* and *C. inatomii* contained a few doublet peaks. The electropherograms of these sequences were carefully inspected by eye, and were unambiguously resolved into known *Plasmodium* lineages.

### Results and discussion

#### *Plasmodium* spp. from mosquitoes in Rinshi-no-mori park

Five mosquito species were collected from Rinshi-no-mori park. *Culex p. pallens* was the most prevalent (*n* = 533 females), followed by *C. sasai* (*n* = 19), *Lutzia vorax* (*n* = 16), *Orthopodomyia anopheloides* (*n* = 9), and *C. rubithoracis* (*n* = 5). All mosquitoes were dissected, and only *C. p. pallens* was found to be infected with malaria parasites.

**Fig. 1:**
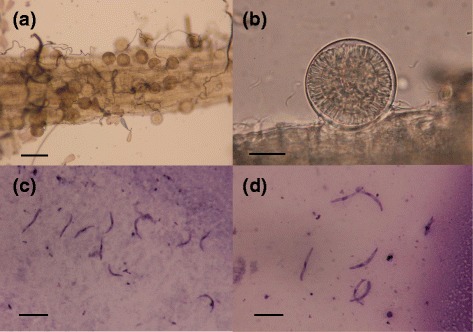
Oocysts (**a**, **b**) and Giemsa-stained sporozoites (**c**, **d**) of avian malaria parasites from *Culex pipiens pallens* and *C. inatomii*. **a**–**c**: CXINA02 [GanBank: AB920777] from *C. inatomii*, **d**: SGS1-*Plasmodium relictum* [AF495571] from *C. p. pallens*. Scale bar,100 μm (**a**), 20 μm (**b**), and 10 μm (**c**, **d**)

Oocysts were observed in the midgut of 21 (3.94 %) *C. p. pallens*. Motile sporozoites were found in the salivary gland of 11 of these specimens (Table 1 and Fig. 1). The overall sporozoite rate (*i. e*., the proportion of mosquitoes with sporozoites) was 2.06 %. This rate is significantly lower (Fisher’s exact test, *p* = 0.001) than the 8.31 % of samples that tested positive for *Plasmodium* DNA in a previous study [13]. All infected mosquitoes also tested positive by PCR, and four cytochrome *b* lineages of avian *Plasmodium* spp. (CXPIP09, GRW4, GRW11 and SGS1) were identified (Table 1). CXPIP09 and SGS1 were the most prevalent, and accounted for >85 % of infections. The dominance of CXPIP09 and SGS1 was consistent with PCR-based studies [13]. However, we did not detect PADOM02 (Table 1), perhaps due to the small sample size or the inability of *C. p. pallens* to support its development. GRW4 and GRW11 that had been previously absent and detected at low frequency, respectively, were found to complete sporogony in *C. p. pallens* (Table 1). Notably, one specimen was co-infected with two different lineages of *P. relictum*, GRW4 and GRW11.

**Table 1:** Avian malaria parasites found from *Culex pipiens pallens* in Rinshi-no-mori park, Tokyo

*Plasmodium* lineage	Previous PCR study [2]		This study: dissection and PCR				Vector status
	(*N* = 1252)		(*N* = 533)				
	DNA	Infection rate (%)	Oocysts	Infection rate (%)	Oocysts & sporozoites	Sporozoite rate (%)	
CXPIP09	43	3.43	6	1.13	3	0.56	Competent
SGS1-*P. relictum*	30	2.40	4	0.75	5	0.94	Competent
PADOM02	16	1.28					Unknown
GRW11-*P. relictum*	3	0.24			1*	0.19	Competent
CXPIP11	1	0.08					Unknown
CXPIP12	4	0.32					Unknown
CXPIP13	1	0.08					Unknown
CXPIP14	1	0.08					Unknown
GALLUS01- *P. gallinaceum*	4	0.32					Not competent [2, 30]
SYAT05-*P. vaughani*	1	0.08					Unknown
GRW4-*P. relictum*					3*	0.56	Competent
Total	104	8.31	10	1.87	11	2.06	

*One *C. p. pallens* specimen was co-infected with CXQUI01 and GRW4-*P. relictum*. GenBank accession numbers: CXPIP09 [AB458850], SGS1-*P. relictum* [AF495571], PADOM02 [DQ058612], GRW11- *P. relictum* [AY831748], CXPIP11 [AB477121], CXPIP12 [AB477122], CXPIP13 [AB477126], CXPIP14 [AB477125], GALLUS01-*P. gallinaceum* [AY099029], SYAT05 [DQ847271] and GRW4-*P. relictum* [AF254975]

Of the four lineages that achieve sporogony in *C. p. pallens*, GRW4, GRW11 and SGS1 belong to the same morphological species, *Plasmodium relictum* [16–18], and are the most widely distributed [15, 19]. For example, SGS1 was found in 62 avian species from Africa, Asia, Europe and Oceania. On the other hand, CXPIP09 has been found exclusively in Japan [15], although its avian hosts are widespread in eastern Asia, such as *Corvus macrorhynchos*, *Passer montanus* [2], *Lanius bucephalus* (KS Kim unpublished data), *Cyanopica cyana*, *Parus minor*, *Treron sieboldii*, and *Zosterops japonicus* (Koichi Murata personal communication). The reason for the limited distribution of CXPIP09 is unknown, as its natural vector, *C. p. pallens*, is also found in the same geographic range as the hosts [20]. Of note, field populations of a competent vector species may vary significantly in susceptibility to the same parasite species, depending on innate immunity and the microbiota in the midgut [21, 22]. Therefore, specific adaptation to *C. p. pallens* in Japan might have stringently limited the distribution of CXPIP09.

#### *Plasmodium* spp. from mosquitoes in Sakata wetland

In Sakata wetland, 4293 female *C. inatomii* were collected, along with *C. p. pallens* (*n* = 459), *C. orientalis* (*n* = 10) and *C. tritaeniorhynchus* (*n* = 6). Of 1314 *C. inatomii* dissected, six specimens had oocysts and sporozoites (Table 2 and Fig. 1). The sporozoite rate (0.46 %) was similar (Fisher’s exact test, *p* > 0.05) to the frequency of *Plasmodium* DNA (0.51 %) in a previous study [11]. All six specimens subsequently tested positive by PCR, and three avian *Plasmodium* lineages (CXINA01, CXINA02 and CXQUI01) were identified, with sporozoite rates ranging from 0.08 to 0.30 %. One specimen was co-infected with CXINA02 and CXQUI01. CXINA02 was a novel lineage, and was deposited in GenBank under accession number AB920777. Two *C. tritaeniorhynchus* and 33 *C. p. pallens* were dissected and none were infected.

**Table 2:** Avian malaria parasites found from *Culex inatomii* in Sakata wetland, Niigata

*Plasmodium* lineage	Previous PCR study [3]		This study: dissection and PCR		Vector status
	(*N* = 7519)		(*N* = 1314)		
	DNA	Infection rate (%)	Oocysts & sporozoites	Sporozoite rate (%)	
CXQUI01	15	0.20	2**	0.15	Competent
CXINA01	9	0.12	1	0.08	Competent
CXPIP10	8	0.11			Unknown
PADOM02	3	0.04			Unknown
CXPIP09	1	0.01			Unknown
SYBOR02	1	0.01			Unknown
GALLUS01-*P. gallinaceum*	1	0.01			Unknown
CXINA02			4**	0.30	Competent
Total	38	0.51	6	0.46	

**One *C. inatomii* specimen was co-infected with CXQUI01 and CXINA02. GenBank accession numbers: CXQUI01 [AB308051], CXINA01 [AB690267], CXPIP10 [AB477128], PADOM02 [DQ058612], CXPIP09 [AB474376], SYBOR02 [DQ368392] and CXINA02 [AB920777]

The current data demonstrates for the first time that *C. inatomii* is a natural vector of avian malaria. Indeed, DNA from seven *Plasmodium* lineages was previously detected in *C. inatomii* from Sakata (Table 2). Of these, three lineages (CXQUI01, CXINA01 and CXPIP10) were the most prevalent, and comprised >86 % of infections in 2007–2010 [11]. Thus, sporogony of CXINA01 and CXQUI01 in *C. inatomii* was not unexpected, but the dominance of the novel lineage CXINA02 was. The difference in dominant lineages in mosquitoes between now and 2007–2010 may reflect changes in the parasite species circulating among host birds.

Unfortunately, the avian host species for CXINA01, CXINA02 and CXQUI01 are presently unknown. The spatial distribution of adult *C. inatomii* is restricted to areas near larval habitats [23, 24], with flight range estimated at <200 m in Sakata [25]. *Culex inatomii* feeds most commonly on *Acrocephalus orientalis*, a summer migratory bird that breeds in the reed fields at Sakata [11]. Hence, *A. orientalis* warrants investigation as a candidate natural host of avian malaria parasites found in *C. inatomii*.

### Conclusions

Microscopic confirmation of sporogony, followed by genetic identification of infecting *Plasmodium* parasites, demonstrated that *C. p. pallens* and *C. inatomii* are natural vectors of four (CXPIP09, GRW4, GRW11 and SGS1) and three (CXINA01, CXINA02 and CXQUI01) lineages of avian *Plasmodium*, respectively. Ideally, transmission to a vertebrate host via a mosquito vector should be experimentally demonstrated [26]. However, such demonstration is difficult for wildlife parasites, usually because of limited availability of natural hosts and vectors. In addition, previous studies have shown that experimental transmission from wild mosquitoes to laboratory hosts (such as poultry in case of avian malaria) is difficult to achieve as well, usually because infected wild mosquitoes are rare and are reluctant to take blood meals under laboratory conditions [27–29]. In light of these, we believe that demonstration of sporogony via a systematic procedure combining dissection and PCR is the most feasible approach to identify natural vectors of wildlife malaria.

## References

[1] WHO (1975). Division of Malaria and Other Parasitic Diseases. Manual on practical entomology in malaria Part II Methods and techniques.

[2] Wharton RH, Eyles DE, Warren M, Cheong WH (1964). Studies to determine the vectors of monkey malaria in Malaya. Ann Trop Med Parasitol.

[3] Wharton RH, Eyles DE, Warren M, Moorhouse DE, Sandosham AA (1963). Investigations leading to the identification of members of the *Anopheles umbrosus* group as the probable vectors of mouse deer malaria. Bull World Health Organ.

[4] Bensch S, Hellgren O, Pérez-Tris J (2009). MalAvi: a public database of malaria parasites and related haemosporidians in avian hosts based on mitochondrial cytochrome *b* lineages. Mol Ecol Resour.

[5] Valkiūnas G (2011). Haemosporidian vector research: marriage of molecular and microscopical approaches is essential. Mol Ecol.

[6] Outlaw DC, Ricklefs RE (2014). Species limits in avian malaria parasites (Haemosporida): how to move forward in the molecular era. Parasitology.

[7] Kim KS, Tsuda Y, Sasaki T, Kobayashi M, Hirota Y (2009). Mosquito blood-meal analysis for avian malaria study in wild bird communities: laboratory verification and application to *Culex sasai* (Diptera: Culicidae) collected in Tokyo. Japan Parasitol Res.

[8] Valkiūnas G, Kazlauskienė R, Bernotienė R, Palinauskas V, Lezhova TA (2013). Abortive long-lasting sporogony of two *Haemoproteus* species (Haemosporidia, Haemoproteidae) in the mosquito *Ochlerotatus cantans*, with perspectives on haemosporidian vector research. Parasitol Res.

[9] Ramsey JM, Beaudoin RL, Bawden MP, Espinal CA (1983). Specific identification of *Plasmodium* sporozoites using an indirect fluorescent antibody method. Trans R Soc Trop Med Hyg.

[10] Santiago-Alarcon D, Palinauskas V, Schaefer HM (2012). Diptera vectors of avian Haemosporidian parasites: untangling parasite life cycles and their taxonomy. Biol Rev Camb Philos Soc.

[11] Kim KS, Tsuda Y (2012). Avian *Plasmodium* lineages found in spot surveys of mosquitoes from 2007 to 2010 at Sakata wetland, Japan: do dominant lineages persist for multiple years?. Mol Ecol.

[12] Kim KS, Tsuda Y, Yamada A (2009). Bloodmeal identification and detection of avian malaria parasite from mosquitoes (Diptera: Culicidae) inhabiting coastal areas of Tokyo bay. Japan J Med Entomol.

[13] Kim KS, Tsuda Y (2010). Seasonal changes in the feeding pattern of *Culex pipiens pallens* govern the transmission dynamics of multiple lineages of avian malaria parasites in Japanese wild bird community. Mol Ecol.

[14] Waldenström J, Bensch S, Hasselquist D, Östman Ö (2004). A new nested polymerase chain reaction method very efficient in detecting *Plasmodium* and *Haemoproteus* infections from avian blood. J Parasitol.

[15] MalAvi: A database for avian haemosporidian parasites Version 2.2.1. http://mbio-serv2.mbioekol.lu.se/Malavi/. Accessed 25 Mar 2015.

[16] Palinauskas V, Kosarev V, Shapoval A, Bensch S, Valkiūnas G (2007). Comparison of mitochondrial cytochrome *b* lineages and morphospecies of two avian malaria parasites of the subgenera *Haemamoeba* and *Giovannolaia* (Haemosporida: Plasmodiidae). Zootaxa.

[17] Valkiūnas G, Zehtindjiev P, Hellgren O, Ilieva M, Iezhova TA, Bensch S (2007). Linkage between mitochondrial cytochrome *b* lineages and morphospecies of two avian malaria parasites, with a description of *Plasmodium* (*Novyella*) *ashfordi* sp. nov. Parasitol Res.

[18] Kazlauskienė R, Bernotienė R, Palinauskas V, Lezhova TA, Valkiūnas G (2013). *Plasmodium relictum* (lineages pSGS1 and pGRW11): complete synchronous sporogony in mosquitoes *Culex pipiens pipiens*. Exp Parasitol.

[19] Hellgren O, Atkinson CT, Bensch S, Albayrak T, Dimitrov D, Ewen JG (2015). Global phylogeography of the avian malaria pathogen *Plasmodium relictum* based on MSP1 allelic diversity. Ecography.

[20] Tanaka K, Mizusawa K, Saugstad E (1979). A revision of the adult and larval mosquitoes of Japan (including the Ryukyu Archipelago and the Ogasawara islands) and Korea (Diptera: Culicidae). Contrib Am Entomol Inst.

[21] Niaré O, Markianos K, Volz J, Oduol F, Touré A, Bagayoko M (2002). Genetic loci affecting resistance to human malaria parasites in a West African mosquito vector population. Science.

[22] Cirimotich CM, Dong Y, Clayton AM, Sandiford SL, Souza-Neto JA, Mulenga M (2011). Natural microbe-mediated refractoriness to *Plasmodium* infection in *Anopheles gambiae*. Science.

[23] Tsuda Y, Sasaki E, Sato Y, Katano R, Komagata O, Isawa H (2009). Mosquito collections from coastal areas of Tokyo Bay receiving migratory birds. Med Entomol Zool.

[24] Tsuda Y, Haseyama M, Ishida K, Niizuma J, Kim KS, Yanagi D (2012). After-effects of Tsunami on distribution and abundance of mosquitoes in rice-field areas in Miyagi Prefecture, Japan in 2011. Med Entomol Zool.

[25] Tsuda Y, Kim KS (2013). Outbreak of *Culex inatomii* in disaster areas of the Great East Japan earthquake and tsunami in 2011, with ecological notes on their larval habitats, biting behavior, and reproduction. J Am Mosq Control Assoc.

[26] Barnett HC, Strouhal H, Beier M (1962). The incrimination of arthropods as vectors of disease. Proceedings of the 11th International Congress on Entomology, Vienna 1960.

[27] Reeves WC, Herold RC, Rosen L, Brookman B, Hammon WM (1954). Studies on avian malaria in vectors and hosts of encephalitis in Kern county, California. II. Infections in mosquito vectors. Am J Trop Med Hyg.

[28] Forrester DJ, Nayar JK, Foster GW (1980). *Culex nigripalpus*: a natural vector of wild turkey malaria (*Plasmodium hermani*) in Florida. J Wildl Dis.

[29] Beier JC, Trpis M (1981). Incrimination of natural culicine vectors which transmit *Plasmodium elongatum* to penguins at the Baltimore Zoo. Can J Zool.

[30] Weathersby AB (1962). Susceptibility of certain Japanese mosquitoes to *Plasmodium gallinaceum* and *Plasmodiun berghei*. J Parasitol.

